# Digital therapeutics from bench to bedside

**DOI:** 10.1038/s41746-023-00777-z

**Published:** 2023-03-10

**Authors:** Changwon Wang, Chungkeun Lee, Hangsik Shin

**Affiliations:** 1 Biomedical Engineering Research Center, Asan Medical Center, Seoul, 05505 Republic of Korea; 2 Digital Health Devices Division, Medical Device Evaluation Department, National Institute of Food and Drug Evaluation, Ministry of Food and Drug Safety, Osong, 28159 Republic of Korea; 3 Department of Digital Medicine, Asan Medical Center, University of Ulsan College of Medicine, Seoul, 05505 Republic of Korea

**Keywords:** Therapeutics, Health care

## Abstract

As a new therapeutic technique based on digital technology, the commercialization and clinical application of digital therapeutics (DTx) are increasing, and the demand for expansion to new clinical fields is remarkably high. However, the use of DTx as a general medical component is still ambiguous, and this ambiguity may be owing to a lack of consensus on a definition, in addition to insufficiencies in research and development, clinical trials, standardization of regulatory frameworks, and technological maturity. In this study, we conduct an in-depth investigation and analysis of definitions, clinical trials, commercial products, and the regulatory status related to DTx using published literature, ClinicalTrials.gov, and web pages of regulatory and private organizations in several countries. Subsequently, we suggest the necessity and considerations for international agreements on the definition and characteristics of DTx, focusing on the commercialization characteristics. In addition, we discuss the status and considerations of clinical research, key technology factors, and the direction of regulatory developments. In conclusion, for the successful settlement of DTx, real-world evidence-based validation should be strengthened by establishing a cooperative system between researchers, manufacturers, and governments, and there should be effective technologies and regulatory systems for overcoming engagement barriers of DTx.

## Introduction

With the development of IT, numerous healthcare technologies using personal smart devices and information have been proposed, and are expected to introduce a paradigm shift in medicine and healthcare. These new healthcare technologies have been provided in the form of health apps and is primarily used to support major medical practices or to promote wellness in prevention and prognosis management. However, as the amount and value of health-related data increase, the demands of consumers and medical service providers for digital medical technology transform from wellness products that have not proven clinically effective to products that have proven clinical effectiveness for the prevention, management, and treatment of diseases. This means digital technology can supplement and replace medical treatment beyond simple bio-signal measurement or health management. From this perspective, digital therapeutics (DTx), treatment techniques based on digital technology, have been defined and studied in earnest since the establishment of Digital Therapeutics Alliance (DTA), which consists of healthcare companies and stakeholders in 2017^1^. DTx is considered as a subcategory of digital medicine, part of a broader category called digital health. Digital health, digital medicine, and DTx products have different requirements for clinical evidence and regulatory oversight because of different claim levels and therefore have different risk levels. DTx with prescription-only characteristics are classified as prescription DTx (PDTx). The primary difference between DTx and wellness applications is that DTx applications are developed with clinical evidence to target specific medical conditions, particularly major chronic diseases^2^.

Commercial DTx that have been released thus far was primarily developed for indications such as chronic and neuropsychiatric diseases which can be treated through behavioral change. DTx are rapidly being commercialized in the United States and Europe^3,4^. However, even for commercialized products, they have not been fully implemented into clinical practice owing to insufficient evidence to prove their effectiveness.

The objective of this study is to propose challenges and recommendations for DTx to be used in clinical settings beyond research and development. We explore trends and characteristics in the major DTx domains through literature and case studies on definitions, intended indications, commercial products, clinical trial studies, and regulations. In addition, we derive major challenges by analyzing the interconnections between research and development, clinical trials, and regulatory domains by comprehensively considering commercial or under-researched DTx. Furthermore, from the results of the investigation and analysis, we suggest essential considerations for each DTx domains and present the discourse required for DTx to be effectively established as a general medical practice in the future.

## Results

### Definition of digital therapeutics

In 2015, Sepah et al. first mentioned the term “digital therapeutics” and expressed that DTx are “evidence-based behavioral treatments delivered online” that can increase healthcare accessibility and effectiveness^5^. The DTA, one of the most active organizations in defining and disseminating DTx, defined DTx as “evidence-based therapeutic interventions that are driven by high-quality software programs to treat, manage, or prevent a disease or disorder”^6^. However, no clear international agreement on the definition exists, and it is being interpreted or applied differently in countries and research institutes. When examining the definitions and responses to DTx in each country, South Korea is the only country that has defined DTx: “software as a medical device that provides evidence-based therapeutic intervention to patients to prevent, manage, or treat a medical disorder or disease”. In other countries, such as the US, Germany, the UK, Japan, Australia, China, and France, DTx are not defined at the government level and are treated as general medical devices, as summarized in Table 1.

**Table 1:** Definition of digital therapeutics by country and institution.

Institute	Definition of Digital therapeutics
European data protection supervisor	Digital therapeutics (DTx) are evidence-based therapeutic interventions driven by software to prevent, manage, or treat a medical disorder or disease. In other words, DTx are patient-facing software applications that help patients treat, prevent, or manage a disease and that have a proven clinical benefit^59^.
Digital therapeutics alliance	Digital therapeutics deliver medical interventions directly to patients using evidence-based, clinically evaluated software to treat, manage, and prevent a broad spectrum of diseases and disorders^6^.
Ministry of Food and Drug Safety, South Korea	Software as a medical device that provides evidence-based therapeutic intervention to patients for prevention, control, or treatment of medical disabilities and/or diseases^26^. ※ The use of digital therapeutics is for patients who require therapeutic intervention

The term DTx is similar to software as a medical device (SaMD) in that they both represent software used for medical purposes. While SaMD is a generic term for software intended to be used for one or more medical purposes, the term DTx is limited to software that intervenes with treatment based on clinical evidence for the treatment, management, or prevention of diseases or disorders. Therefore, most public, or regulatory authorities treat DTx as a sub-concept of SaMD without a special regulatory category.

### Characteristics of digital therapeutics

Table 2 shows a comparison of the characteristics of pharmacotherapy and DTx. A common characteristic of conventional pharmacotherapy and DTx is that the therapeutic effect for a specific disease must be clinically verified, and a prescription is required; however, they have differences in all stages from development, clinical trial, regulatory approval, distribution, clinical application, and post-marketing management. DTx require minimal development cost compared with pharmaceuticals (<1%), and the development period is approximately half^7^. In addition, because they are not consumed due to use, manufacturing facilities and material costs for additional production after initial development are not required. Even for the distribution channels, unlike pharmaceuticals delivered to patients through manufacturers, wholesalers, retailers, and medical suppliers, DTx simplify the delivery path to patients through developers and platform providers.

**Table 2:** Comparison between conventional pharmacotherapy and digital therapeutics.

Category		Pharmaceutical	Digital therapeutics
Commonalities		Clinically proven therapeutic effect and prescription for specific diseases	
Differences	Development cost	High (about $1.8B)^60^	Relatively low
	Development period^7^	Average 12 years	Average 3 to 7 years
	Manufacturing	Continuous production required through manufacturing facilities	No additional manufacturing required after initial development
	Distribution channel	Manufacturer – wholesaler – retailer – medical service supplier (hospital, clinic, pharmacy, etc.) - Patient	Developer – Platform provider – Patient
	Phases of clinical trials	Human pharmacology study (Phase 1) Exploratory study (Phase 2) Confirmatory study (Phase 3)^8^	Exploratory study (Pilot, optional) Confirmatory study (Pivotal)^9^
	Regulation	Pharmaceutical law	Medical device law
	Medication monitoring	Manual monitoring	Real-time automatic monitoring^12^
	Side effects^1^	Drug toxicity	Minor side effects owing to the use of mobile devices
	Medication adherence	50%^11^	80%^10^
	Maintenance	Unavailable	Available through update
	Withdrawal and disposal	User	Provider
	Expiration date	The final day that the manufacturer guarantees	Depending on the efficacy changing over time
	Classification	OTC drug	DTx
		ETC drug	PDTx
	Efficacy influencing factors^12,13^	Primarily physiological and demographic factors	In addition to demographic factors, it is also affected by sociocultural and cognitive abilities.
	Accessibility by patient	Accessible without education	Need prior education
	Prerequisites	Not required	Digital device, Appropriate level of cognitive ability,
	Prescription	Mandatory (ETC only)	Mandatory (PDTx only)
	Data security	Not applicable	Need cyber security solution
	Clinical challenges^14^ (i.e., psychopharmacology)	Blood–brain barrier (Neurology)	Patient engagement

*SaMD* software as medical device, *ETC* ethical the counter, *OTC* over the counter, *DTx* digital therapeutics, *PDTx* prescription DTx.

For clinical trials, unlike pharmaceuticals that require phase-3 clinical trials that include drug safety and pharmacokinetic evaluation, DTx can obtain marketing approval through piloting (optional) and pivotal clinical trials through the medical device regulatory pathway in most countries^8,9^. Additionally, DTx only provide software-based interventions; thus, unlike existing pharmacotherapy that has side effects due to drug toxicity, they are considered to have only minor side effects owing to the use of mobile devices^1^. Medication adherence of DTx is known to be approximately 80%^10^, which is higher than that of pharmacotherapy (50%)^11^. In post-marketing management, the expiration date of pharmaceuticals is determined according to the denaturation of substances, but the expiration date of DTx depends on the efficacy that changes over time. In classification, pharmaceutical drugs are classified as over the counter or ethical the counter drugs depending on whether a prescription is required. Similarly, some of DTx are classified as a PDTx if it requires a prescription. Moreover, DTx can improve their function (efficacy) or discard it through updates. In pharmacotherapy, individual physiological characteristics are the most important influencing factors in determining drug efficacy. However, the important difference is that the efficacy of DTx can be affected not only by demographic factors but also by sociocultural and cognitive abilities^12,13^. The main disadvantages of DTx compared to pharmaceuticals are low patient accessibility from a digital barrier, need for prerequisites, and data security issues. Since DTx operate on a digital platform, it is essential to understand digital devices; therefore, education prior to use is required, and it can be applied only to patients who have digital devices and some level of cognitive ability. In addition, since DTx store user data in digital form, there is a risk of leakage of sensitive personal data; therefore, additional preventative cyber security is required. The clinical challenge of pharmacotherapy is considered as a blood–brain barrier in drugs for neurological disease; however, for DTx, patient engagement is considered a major factor in determining the success of treatment in the future as it presupposes active participation of the patient^14^.

### Clinical trials of digital therapeutics

After screening 50,872 investigated clinical literature based on duplication, year of publication, non-journal article, and research scope, forty-five clinical trials were finally analyzed, excluding cases without National Clinical Trial (NCT) number or meta-analysis; of these, thirty-one were registered with clinicaltrials.gov and fourteen were presented in DTA website. A flowchart of the clinical trial search process is shown in Supplementary Figs. 1, 2, 3, and 4, and the NCT-numbered clinical trials related to DTx are summarized in Supplementary Table 1.

Detailed clinical trials related to psychiatric indications showed the largest number at 31.1%, neurological at 22.2%, endocrine at 20%, respiratory at 11.2%, poisoning at 8.9%, and cardiovascular disease at 6.7%. For clinical studies currently in progress, neuropsychiatric and chronic diseases accounted for the majority of indications; however, it has been shown that the scope of DTx is expanding with indications for neurological diseases as follows. Neurodegenerative diseases such as Parkinson’s disease, mastectomies, cancer diseases such as lump resections, blood diseases such as multiple myeloma, solitary plasmacytoma, plasma cell diseases such as amyloidosis, multiple sclerosis, multiple sclerosis-related depression and anxiety, fibromyalgia, back pain, chronic diseases such as heart failure (different from existing chronic diseases), autism spectrum disorders, schizophrenia, major depressive disorder, heart failure, pain, acute postoperative pain, systemic diseases such as lupus erythematosus, and dysarthria after stroke^15,16^.

For national clinical trial registration, the United States registered the most with 27 cases (60%), followed by Switzerland with two (4.4%), Finland with three (6.7%), South Korea with two (4.4%), Italy with two (4.4%), Brazil with one (2.2%), Malaysia with one (2.2%), Singapore with one (2.2%), Canada with one (2.2%), Israel with one case (2.2%), and Poland with one case (2.2%). three case (6.7%) had no clinical trial information on the ClinicalTrials.gov website. Clinical research was conducted by various institutions, including 24 companies (53.3%), four pharmaceutical companies (8.9%), seven universities (15.6%), four hospitals (8.9%), three research institutes (6.7%), two medical consortiums (4.4%), and one other (2.9%).

**Fig. 1:**
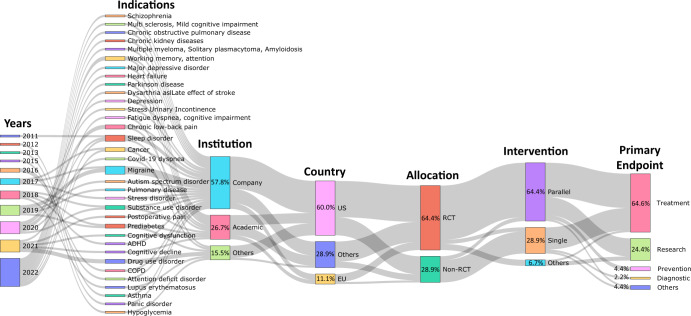
Sankey diagram depicting digital therapeutics-related clinical research studies that have been assigned a National Clinical Trial (NCT) number. A Sankey diagram was utilized to analyze trends in indication, institution, country, trial type, intervention, and major outcomes. Between 2010 and 2022, a total of 31 clinical trials related to digital therapeutics were identified on the ClinicalTrials.gov website using the keyword “Digital therapeutics”. The clinical trials were conducted by a variety of institutions, including pharmaceutical companies, hospitals, research institutes, medical consortia, universities, and corporations. These trials were carried out in multiple countries, including the United States, Europe, Switzerland, Finland, South Korea, Italy, Brazil, Malaysia, Singapore, Canada, Israel, and Poland. The clinical trials employed interventions such as parallel, single, crossover, and factorial designs, with primary objectives that included treatment, research, prevention, diagnosis, supportive care, and basic science.

Figure 1 shows a Sankey diagram analyzing clinical studies registered on ClinicalTrials.gov or on DTA website, or clinical studies that are not registered on ClinicalTrials.gov but presented through research papers. This diagram shows the relationship between clinical trial registration or publication timing, indications, sponsor type, country, clinical trial type, study design method, and primary outcome. Figure 1 shows that the types of indications have been diversifying over the years, showing that companies are prioritizing clinical trials more proactively. In addition, more than half of clinical trials were conducted in the US. Most clinical trials were conducted with randomized controlled trials (RCTs). For clinical study design, parallel had the highest proportion. The primary outcome included all of the general medical practice areas such as treatment, diagnosis, and prevention, but the proportion of treatment was the highest, followed by research. The proportion of other primary outcomes was insignificant.

### Commercialization of digital therapeutics

Many countries are promoting the commercialization of DTx through multi-sector cooperation with regulatory authorities, pharmaceutical companies, and medical experts. In the initial stage, DTx are approved and commercialized primarily for chronic diseases such as diabetes, cardiovascular disease, respiratory disease, and chronic pain or neuropsychiatric diseases such as drug addiction, sleep disorder, schizophrenia, chronic pain, and attention deficit hyperactivity disorder (ADHD)^3^. However, DTx for more diverse indications, such as irritable bowel syndrome^17^, migraine^18^, hip and knee replacement surgery, and ear disease, have been launched recently. Most DTx have been released through Food and Drug Administration (FDA) clearance led by the US (see Table 3), and currently, CE marked DTx products are being launched in Europe, primarily in Germany and Belgium^1^.

**Table 3:** FDA cleared DTx products and regulatory status.

Product	Manufacturer	Indication	Pathway	Nomenclature	Regulatory Class	Prescription	Clinicals outcome in premarket stage	Approval year
Dario	LabStyle Innovations	Type 1,2 diabetes	510(k)	System, test, blood glucose, over the counter	Class II		O	2015
Insulia	Voluntis	Type 1,2 diabetes	510(k)	Calculator, drug dose	Class II	O		2016
reSET	Pear Therapeutics	Substance use disorder	De novo	Computerized behavioral therapy device for psychiatric disorders	Class II	O	O	2016
Natural Cycles	Natural Cycles	Birth control	De novo	Software application for contraception	Class II		O	2017
MindMotion Go	Mindmaze	Neurorehabilitation	510(k)	Measuring exercise equipment	Class II	O		2017
My Dose Coach	Sanofi	Type 1, 2 diabetes	510(k)	Calculator, drug dose	Class II	O		2017
reSET-O	Pear Therapeutics	Opioid use disorder	510(k)	Computerized behavioral therapy device for psychiatric disorders	Class II	O	O	2018
Freespira	Palo Alto Health Sciences	Post-traumatic stress disorder	510(k)	Biofeedback Device	Class II		O	2018
Propeller	RESMED (Propeller Health)	Chronic obstructive pulmonary disease	510(k)	Nebulizer	Class II		O	2018
TALi Train	TALI Digital	Attention impairment	510(k) exempt	Computerized cognitive assessment aid	Class II			2018
leva	Renovia, Inc	Urinary incontinence	510(k)	Perineometer	Class II	O		2018
d-Nav	HYGIEIA	Type 1,2 diabetes	510(k)	Calculator, drug dose	Class II	O		2019
Somryst	Pear Therapeutics	Chronic Insomnia	510(k)	Computerized behavioral therapy device for psychiatric disorders	Class II	O	O	2019
WellDoc	BlueStar	Type 1,2 diabetes	510(k)	Accessories, pump, infusion	Class II	O		2020
EndeavorRx	Akili Interactive Labs	Pediatric attention deficit hyperactivity disorder	De novo	Digital therapy device for attention deficit hyperactivity disorder	Class II	O	O	2020
Nerivio	Theranica	Migraine	510(k)	Trunk and limb electrical stimulator to treat headache	Class II		O	2020
Nightware	NightWare	Post-traumatic stress disorder	De novo	Digital therapy device to reduce sleep disturbance for psychiatric conditions	Class II	O	O	2020
Parallel	Mahana Therapeutics	Irritable bowel syndrome	De novo	Computerized behavioral therapy device for treating symptoms of gastrointestinal conditions	Class II		O	2020
RelieVRx	AppliedVR	Pain relief	De novo	Virtual reality behavioral therapy device for pain relief	Class I	O		2021
Mahana	Mahana Therapeutics	Irritable bowel syndrome	510(k)	Computerized behavioral therapy device for treating symptoms of gastrointestinal conditions	Class II	O		2021

A representative example of commercialized DTx is reSET (Pear Therapeutics Inc., MA, USA). reSET is the first interactive FDA cleared DTx for cognitive-behavioral therapy of drug and alcohol addiction patients. reSET provides a professional online counseling service and a face-to-face treatment service with medical staff according to the results of a patient self-questionnaire. In addition, EndeavorRx (Akili Interactive Labs Inc., MA, USA), developed as a DTx for pediatric ADHD, demonstrated that it can improve a patient’s attention index (API) by stimulating and activating the prefrontal cortex through video games^19^, and FDA-510(k) clearance and CE mark were obtained. Teva Pharmaceuticals’ Propeller Health (ResMed (Propeller Health), WI, USA)^20^, and ProAir Digihaler (Teva Pharmaceuticals Inc., NJ, USA)^21^, which are medication reminders with an inhaler, have proven to have a 79% reduction in inhaler use when applied to asthma and COPD patients, respectively, and obtained FDA-510(k) certification^22^. In addition, Sleepio (Big Health, CA, USA), developed for the improvement of sleep disorders, showed that the treatment effect can be improved from 20 to 76% through the sleep management function and online sleep disorder counseling program^23^.

Unlike general medicines, DTx operate by providing treatment content through computer or mobile applications. Because implementation technologies such as web or mobile app development and server construction do not have much differentiation for each indication, the specific treatment effect is determined by the content and application method provided. Therefore, each company’s DTx pipeline will depend on how the company will provide indication-specific content to the DTx platform it has already secured. Development of DTx contents for each indication is primarily performed through the company’s own development or in cooperation with research institutes such as universities or hospitals, and DTx are expanded to various indications by continuing cooperation with existing partnerships or establishing new partnerships with specialized institutions.

**Fig. 2:**
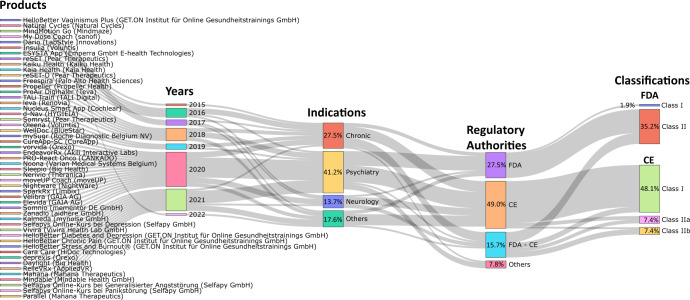
Sankey diagram depicting the major indications and regulatory status of commercial digital therapeutics. Sankey diagrams were employed to analyze trends in the types of indications, regulatory agency types, and class types for commercial digital therapeutic devices. The investigation focused on products listed on the DTA, DiGA, and mhealthbelgium websites, as well as products that had received FDA approval listed in the FDA medical device database. Regulatory authorities for each product were also investigated. Commercial digital therapeutics are primarily launched for indications related to chronic conditions, psychiatry, and neurology, with additional products available for urinary incontinence, ear disorders, hip and knee arthroplasty, tinnitus, irritable bowel syndrome, and vaginismus. Most commercial products have received approval from the FDA and CE regulatory agencies, while others have been approved by Japan’s Ministry of Health, Labour and Welfare (MHLW) and the Medicines and Healthcare products Regulatory Agency (MHRA).

Figure 2 shows a Sankey diagram of commercialized DTx products, year of release, indication, regulatory authority, and regulatory class. The number of commercial products released has a repeating pattern of an increase and a decrease every two years since 2015, but shows an overall increase. Many of the products initially released were related to chronic or neurological diseases, and products released after 2020 were observed to be related to psychiatric disorders. By regulatory authority, most products are FDA cleared and CE marked. A total of 51 products received CE mark or FDA clearance, of which the number of CE marked products (26) was approximately double the number of FDA cleared products (14), and 14 products obtained both FDA clearances and CE marks. Most of the FDA cleared products obtained Class II grade certification, whereas most CE marked products were certified as Class I grade (Supplementary Table 2). However, it is unreasonable to generalize that CE legislation is stricter than FDA legislation; therefore, there are many approvals of low-grade medical devices. Recently, the EU has strengthened regulations from MDD to MDR, and one questionnaire to industry found that 89% of respondents now prefer US rather than EU market entry for innovative devices due to the increased predictability of regulatory requirements^24^. The European DTx approval flow is expected to change after 2021, as the difficulty of obtaining new product approvals has increased, and strict post-marketing surveillance is involved.

### Regulation of digital therapeutics

Six guidelines and one policy document were investigated for DTx-related regulations within the International Medical Device Regulators Forum (IMDRF) member jurisdictions. The FDA has stated that it will permit the distribution and use of devices during public health emergencies without filing a premarket notice under section 510(k), as the FDA considers that DTx do not pose undue risk^25^. Six guidelines were published by the Ministry of Food and Drug Safety (MFDS, South Korea). The guidelines published in 2020 provided guidance on the definition of DTx and documents required to be submitted when obtaining DTx approval in the Korean regulatory system^26^. Subsequently, five guidelines published from 2021 to 2022 suggest safety and performance evaluation methods for DTx for alcoholism^27^, depressive disorder^28^, insomnia^29^, nicotine use disorder^30^, and panic disorder^31^ and provide design examples of clinical trial protocols with primary endpoints, sample size, and hypothesis for each guideline. Although there are no specific guidelines, except in Korea, the continued cases of approval through regulatory authorities in the United States, Europe, and Japan suggest that approval is possible within the current regulatory system for general medical devices. However, researchers are suggesting the establishment of a regulatory system suitable for DTx. Researchers argue that the regulatory system of DTx is considerably vague^32^ or insufficient^33^, and Vilardaga et al. indicated the limitation that DTx regulation does not guarantee usability and continuous adoption^14^. A stakeholder study on the development of the DTx industry reported that it is important to develop guidelines for permits and prepare a simplified regulatory system following research funding, suggesting that the role of the government is important^34^. In addition, researchers urged the development of an internationally harmonized regulatory model to improve the safety and quality of DTx^35–37^.

## Discussion

DTx are classified as medical devices representing regulated products worldwide, and refers to a type of medical device, not general medical devices such as artificial intelligence or implantable medical devices. The definition of DTx is dependent on the regulatory decisions of each country’s authorities, and each authority must follow the medical device nomenclature. However, as suggested in the results regulatory agencies have no formal agreement on the type of DTx as a medical device, and it is defined in various ways by country or institution using similar concepts and keywords.

As discussed above, keywords encompassing various definitions of DTx are summarized with application software, evidence-based medical intervention, preventing, managing, or treating diseases. Combining the above keywords, DTx can be defined as “software which provides evidence-based medical interventions for disease or disorder prevention, management, and treatment.” From the above definition, we derived the following requirements for DTx. First, DTx should be able to provide medical interventions for disease or disorder prevention, management, and treatment. Second, DTx should provide accessibility suitable for the user’s situation-appropriate methods such as application software. Third, for evidence-based treatment, the effectiveness of use must be proven through systematic clinical trials.

To determine whether these requirements have been achieved, agreed criteria for medical intervention effects, software availability, and clinical trial conditions that provide evidence are required, but no clear document has been presented yet. For example, as the use of artificial intelligence technology in medical devices increased, the IMDRF developed a technical document on key terms and definitions of artificial intelligence medical devices for regulatory use^38^. Therefore, regulators should carefully observe the DTx industry, and if the maturity level and terminology between countries are inconsistent, they should consider an agreement on key terms and definitions among international regulators. In addition, if the number of DTx approvals for a specific indication increase, it is necessary to develop standards for safety and efficacy verification through international standards development organizations such as the IEC or ISO, focusing on proven market maturity. Moreover, if the clinical trial protocol for efficacy evaluation for a specific disease group is standardized and the differences by race are small, the clinical trial conditions may be specified within the standard, such as using a medical device with a clearly defined clinical trial method (e.g., pulse oximeter^39^). Finally, DTx-related nomenclature was added to the official international nomenclature of medical devices referenced by various regulatory authorities, such as the global medical device nomenclature (GMDN); thus, DTx products approved under new nomenclatures in each country, e.g., under the FDA de novo pathway, should be available as per internationally harmonized nomenclatures for use.

Since DTx are primarily provided through smart devices, they can be closely related to the user’s daily life and perform continuous active interventions. Owing to these characteristics, DTx have been developed for the main objective of chronic disease management, drug abuse prevention, sleep management, and psychological and psychiatric disease management and treatment that require continuous interaction^4^. Chronic diseases include major diseases such as diabetes, asthma, chronic pain, chronic heart disease, and substance abuse disorders cover lifestyle diseases such as alcoholism, smoking and, medical drug abuse such as opioids. In addition, psychiatric disorders include a wide range of indications, from depression or anxiety disorders to post-traumatic stress disorder, schizophrenia, and ADHD. For sleep, they primarily address sleep disorders such as insomnia. However, recent DTx and ongoing clinical research cases show attempts to make DTx more disease-focused in various clinical fields^16^.

The clinical field application of DTx is expanding to tumors, cranial nerve, obstetrics and gynecological, urinary system, digestive system, orthopedic, respiratory system, immune, otolaryngology, and infection diseases^15^. It also includes a wide variety of indications such as mastectomy, lumpectomy, amyloidosis, multiple myeloma, solitary plasmacytoma, gynecological pain, urinary disorder, migraines, Parkinson’s disease, irritable bowel syndrome, Lupus erythematosus, multiple sclerosis, dyspnea genitopelvic pain/penetration disorder, and cognitive dysfunction. We observed no studies which clearly defined the commonalities of the new fields under consideration for the application of DTx. However, in the role or usage pattern, if it is possible to directly perform cognitive-behavioral intervention or as an auxiliary/complementary material to aid a doctor’s treatment or follow-up management, it tends to be introduced preferentially. Sim et al. predicted that DTx would be preferentially introduced in fields in which cognitive-behavioral interventions are possible and medical demand exceeds supply^40^. Supplementary Table 3 categorizes and shows indications of representative DTx that have been commercialized or are in the research stage.

Most of the DTx developed thus far are provided in the form of smartphone or web-based applications, and there are differences in core technology elements according to the type of indication and target end-user type (Supplementary Table 4). For example, diabetes DTx for diabetes provide treatment/guidance through the mobile web, and DTx related to respiratory diseases such as asthma and chronic obstructive pulmonary disease (COPD) includes measurement functions using Internet-of-Things devices in addition to mobile apps. Furthermore, DTx for pediatric ADHD, eye disease, and psychiatric indications provide virtual reality (VR) devices, game content, and programs for mobile or PC. Therefore, most of the core technologies of DTx developed thus far focus on mobile and web apps. This is because user accessibility to smartphones is very high and monitoring and feedback related to the treatment is easy, which is advantageous for improving medication adherence and maximizing the effect of cognitive-behavioral therapy.

Content is a core technical element of DTx and has various forms such as questionnaires, face-to-face monitoring, games, and virtual reality^41,42^. Content plays an important role in determining the therapeutic effect of DTx; to induce patients to take continuous medication (participation) and increase the therapeutic effect, the provisional content and method are determined by considering the target indication, patient’s age, and sociocultural characteristics. Content is often developed based on the experience and expertise of clinicians; however, recently, cases of applying artificial intelligence to treatment contents or treatment guides have been reported^43,44^, thus continuous attention is required.

The important requirements for efficient DTx are digital adequacy aspects such as the user’s ability to use digital devices and cognitive level. Since DTx are mostly provided in the form of content through digital platforms, the higher the user’s ability to use the digital device, the better the understanding and concentration on the provided content, and the higher the efficacy of the manufacturer’s intention. However, these characteristics of DTx can cause a digital health gap due to digital disparities; therefore, a design process considering patient equity is required. Additionally, to secure the therapeutic effect of DTx, patient engagement is key^13,14^, and can be affected by sociocultural background and demographic characteristics^12,13^. The fact that DTx are influenced by demographic or sociocultural characteristics is not sufficiently empirically proven. However, from the examples of common digital media, we can infer that users’ influence on digital content varies according to external factors. A study which analyzed the factors affecting digital media user acceptance indicated that a reader’s awareness, interest, and intention to use e-books are affected by the reader’s age, education level, and income^45^. It has been reported that digital disparities or e-service discrepancies may occur depending on education level or age in the EU^46^ and income, education level, residence type, and age in Canada^47^. In addition, it has been reported that the ability to use a mobile health app is also related to age, education level, and e-Health literacy^48^. Since DTx are a type of digital content, we can easily infer that the above factors directly affect DTx adequacy.

The above suggests that demographic and sociocultural characteristics of individuals such as age, gender, culture, cognitive ability, digital device usability, social status, and even religion and values should be considered when conducting DTx clinical trials or applying regulations. Akili Interactive Labs, inc.’s DTx for pediatric ADHD is an example of this. Akili’s pediatric ADHD treatment platform, Selective Stimulus Management Engine (SSME), is divided into 8–12, 13–17, and 18 years or older products in the United States, and clinical trials and approval procedures are in progress for each product. However, it has already entered the market without age restrictions in Europe, and clinical trials are being conducted in Japan for 6–17-year-old patients. Although Akili’s pediatric ADHD DTx have a similar platform and configuration, the clinical and approving procedures differ for each country because the platform is localized for language and culture. This is because the degree of the friendliness of the digital platform, the user’s cognitive level, and the sociocultural background can affect the therapeutic effect of the DTx. Lastly, changes in values, culture, and customs according to the change in era can change patients’ perception to the same digital content, which can lead to changes in the efficacy of DTx. Although this cannot be standardized, it means that the efficacy of DTx will naturally change over time. This suggests that there is contemporaneity in the efficacy of DTx. Consequently, DTx require periodic verification even after approval due to the change in efficacy over time, which means that DTx have an expiration date.

Nearly 80% (76.3%, 29 of 38) of the DTx-related clinical trials investigated in this study were RCTs. RCTs are still the best type of evidence that scientists and regulators can agree for validating the efficacy and safety of a device^14^, demonstrating that DTx products are rigorously tested in the clinical phases. A major challenge in current DTx clinical trials is the establishment of appropriate control groups for multinational trials and RCTs. Among the clinical trials investigated in this study, no cases of multinational clinical trials were found, and clinical trials in Asia were also rare. In addition, DTx as software can appear in various forms compared with placebos of pharmaceuticals, and maintaining complete blind conditions is difficult^12,49^; thus, an RCT framework considering the characteristics of DTx should be designed.

Although DTx are approved through RCTs, they are high-risk medical devices (Class III), and regulatory post-approval studies are not compulsory. Moreover, not all licensed DTx satisfy the statutory criteria for post-market surveillance^50^. An RCT is important and necessary to understand the efficacy of treatment; however, real-world data can also complement RCTs by evaluating the generalization of interventions and outcomes in real-world implementations^51^. Therefore, it appears that post-marketing research to observe whether newly approved DTx maintain safety and efficiency in the real-world should be continued through manufacturers and academia.

Although the IMDRF regulatory authority has not yet issued any clear guidelines, DTx are classified as SaMD and approved in the same manner as general medical devices^36^. In Germany’s DiGA-related Fast-Track Procedures^52^, DiGA is applied only to products which have obtained approval in accordance with the European Medical Device Directive (MDD)/European Medical Device Act (MDR); therefore, we consider the permission of DTx is sufficiently possible in the current regulatory system. However, DTx manufacturers are not traditional medical device companies familiar with medical device regulations, and regulatory systems and documents are also not familiar to researchers. Therefore, government efforts such as the publication of guidelines or online seminars to aid DTx manufacturers understand the regulatory system are required.

This study had the following major limitations. First, since this study was conducted only with publicly available data, the current development stage research, clinical trials in preparation, and regulations and guidelines under development by countries and institutions, were not included in the analysis. In addition, because published studies, clinical trials, and commercialized products tend to be reported based on the results of successful implementations, case studies or cause analysis of failure were not conducted. The literature on clinical efficacy verification in the real world was also excluded from the scope because of insufficient cases for each product. The real-world evidence (RWE) of DTx is also used as the major evidence data in determining reimbursement through health technology assessment. Not addressing the reimbursement of DTx is another important limitation of our study. Reimbursement is the biggest stumbling block to dissemination of innovative medical devices, and there are few cases such as DiGA in Germany and Improving Access to Psychological Therapies (IAPT) in UK. However, although the Access to Prescription Digital Therapeutics Act has entered the legislative stage in the US^53^, there have not been sufficient cases to compare and analyze payment schemes because Act has not implemented yet. Therefore, such cases should be considered in future studies.

After successful commercialization in the field of cognitive-behavioral therapy, DTx are expanding their scope of application to various clinical indications and are creating a new ecosystem by linking legacy healthcare and digital platforms. DTx have advantages in terms of not only access to global smart technology, cost-effectiveness, and patient-specific treatment but also response to diseases such as chronic, psychiatric, and neurological diseases that have not been properly managed within the conventional medical system. Therefore, the market value, expectations, and demand for DTx are expected to increase daily. For DTx to progress from the introduction stage and become a successful future treatment technology, barriers such as RWE-based verification should be solved through strengthening the link between research and development, clinical trials, and regulatory domains, establishing strategies to overcome engagement barriers, and preparing efficient regulatory processes. Moreover, infrastructure and clinical adaptability of DTx should be considered for a stable settlement of DTx in clinical areas. Hence, a close cooperation process between researchers, manufacturers, and government should be established. In addition, measures to secure technical and institutional solutions to prevent deepening health disparities and equity in the application owing to digital or medical disparities between countries should be considered from the development stage. Therefore, discussions and agreements centered on various international organizations, including those of medical devices, are required.

## Methods

### Literatures

Research literature, registered clinical trials, regulatory-approved commercial products, and regulations related to DTx were investigated and analyzed, and background data for each area were collected in the following way. First, for research literature, papers published between 2010 and 2022 were investigated from a total of five databases: PubMed, Institute of Electrical and Electronics Engineers (IEEE), Google Scholar, ScienceDirect, and Web of Science. The search string used was as follows: (Digital therapeutics OR DTx) AND (FDA OR SaMD OR MDD OR MDR) AND (Digital health OR Healthcare) AND (Smartphone OR Application). The literature search was conducted only on articles found through the search string, and reviewed articles that were not published in English were included. For scientific articles, non-journal articles were excluded from the analysis.

### Clinical trials

Clinical trial studies through research articles were investigated using the same search string as the literature search. For clinical trial research through the DTA website (https://dtxalliance.org), the contents of the clinical overview described in the detailed description of the product library among DTA website were referred to. In addition, cases were reviewed by searching for the keywords “digital therapeutics” on the ClinicalTrials.gov website. Finally, cases without an NCT number related to clinical studies investigated were excluded from the analysis. All clinical trials investigated only those registered or presented between 2010 and 2022.

### Commercial products

Commercial products included in the DTx-related literature or products registered in the FDA Medical device database^54^ in the product library of the DTA website^55^, as well as products that have received an CE mark in Germany and Belgium^56,57^, were primarily investigated.

### Regulations

Regulatory areas can be divided into premarket approval, which approves and manages medical devices, quality management systems, and post market control^58^. It may include reimbursement for DTx use through insurance in the aftermarket. In this study, the search focused on guidance related to premarket and other regulatory areas were excluded. The reason focusing on guidance is that DTx are part of software as medical devices, and regulatory authorities treat certain categories of medical devices different. We selected the Therapeutic Goods Administration (TGA, Australia), Brazilian Health Regulatory Agency (ANVISA, Brazil), National Medical Products Administration (NMPA, China), Health Canada (HC, Canada), European Commission Directorate (EC, EU), Pharmaceutical and Medical Devices Agency (PMDA, Japan), Ministry of Health of Russian Federation (MHRF, Russia), Health Sciences Authority (HSA, Singapore), MFDS (Republic of Korea), Medicines and Healthcare products Regulatory Agency (MHRA, United Kingdom), and US FDA (United States), the regulatory authorities of the IMDRF member countries, as the target of our investigation, and searched related data on the English website of each regulatory authority. Among the EU members, Germany, which established the Digital Health Apps program (DiGA), was additionally investigated, and Korean homepages were also investigated considering the linguistic accessibility of the authors. The research was conducted by searching the website of each institution with the keywords “Digital therapeutics” OR “DTx” OR “Guidance” OR “Guideline” OR “Policy.” In addition, literature and paper research on regulations proposed by researchers for DTx were conducted based on the same investigation conditions (five databases, investigation period) and “Digital therapeutics” OR “DTx” AND “Regulation” was used as the search string. In the regulatory literature search, only cases in which DTx was clearly mentioned in the scope, keywords, or main contents of the document were included, and cases in which it was described only in broad terms (e.g., software as a medical device, SaMD, software or program) were excluded from the analysis. Flowcharts for the search strategy are presented in Supplementary Figs. 1, 2, 3, 4.

### Reporting summary

Further information on research design is available in the Nature Research Reporting Summary linked to this article.

## Supplementary information

Supplementary Information

REPORTING SUMMARY

## Data Availability

The main data supporting our results in this study are available in the manuscript and Supplementary Information.

## Code availability

No code was used in this study.
