# Supplementary material for: Digital therapeutics from bench to bedside
Source: NPJ Digit Med. 2023 Mar 10;6:38. doi: 10.1038/s41746-023-00777-z (PMC10006069; doi:10.1038/s41746-023-00777-z)
Supplement: Supplementary file 1 — Supplementary Information [file 41746_2023_777_MOESM1_ESM.pdf]

Supplementary Information

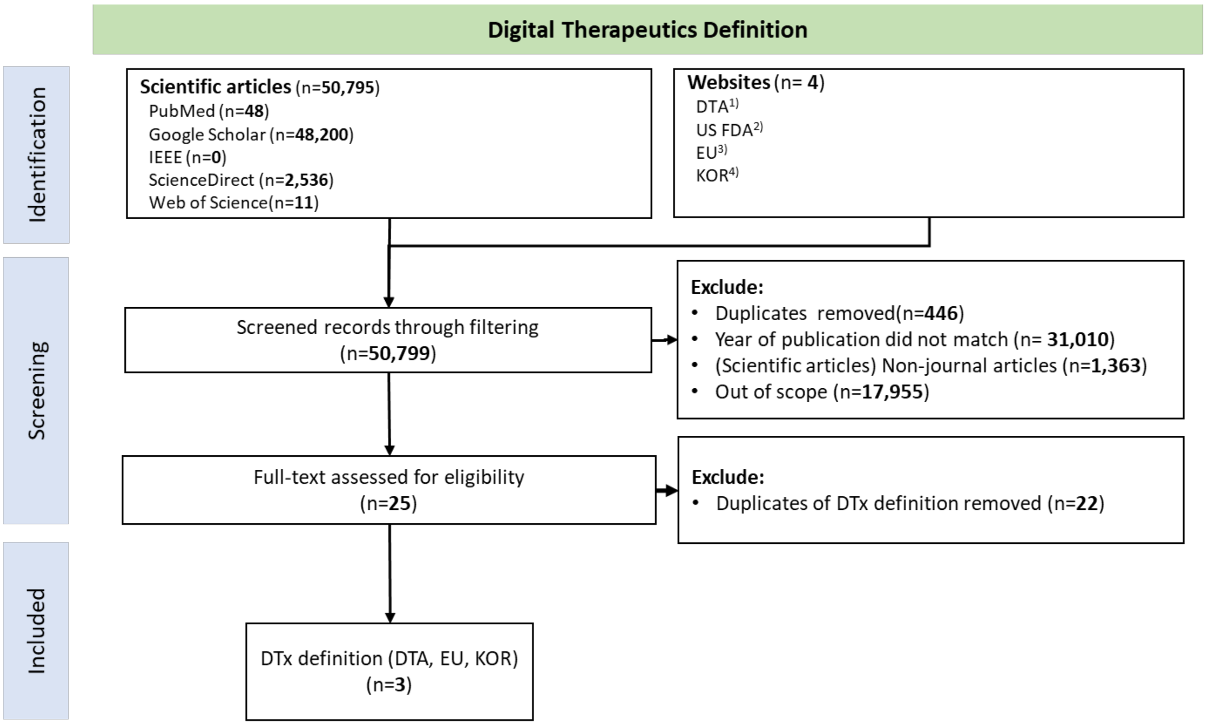

<Search string for scientific articles>
(Digital therapeutics OR DTx) AND (FDA OR SaMD OR MDD OR MDR) AND (Digital health OR Healthcare) AND (Smartphone OR Application)

<Website list>

1) DTA	https://dtxalliance.org/understanding-dtx/what-is-a-dtx/#understand
2) FDA	https://www.fda.gov/medical-devices/digital-health-center-excellence/software-medical-device-samd
3) EU	https://edps.europa.eu/press-publications/publications/techsonar/digital-therapeutics-dtx_en
4) KOR	https://www.mfds.go.kr/eng/brd/m_40/view.do?seq=72624&srchFr=&srchTo=&srchWord=&srchTp=&itm_seq_1=0&itm_seq_2=0&multi_itm_seq=0&company_cd=&company_nm=&page=1

<Abbreviation list>
DTA : Digital Therapeutics Alliance, **US FDA** : U.S. Food and Drug Administration, **EU** : European Union, **KOR** : Republic of Korea

Supplementary Figure 1. Flowchart for search process related to definition of digital therapeutics

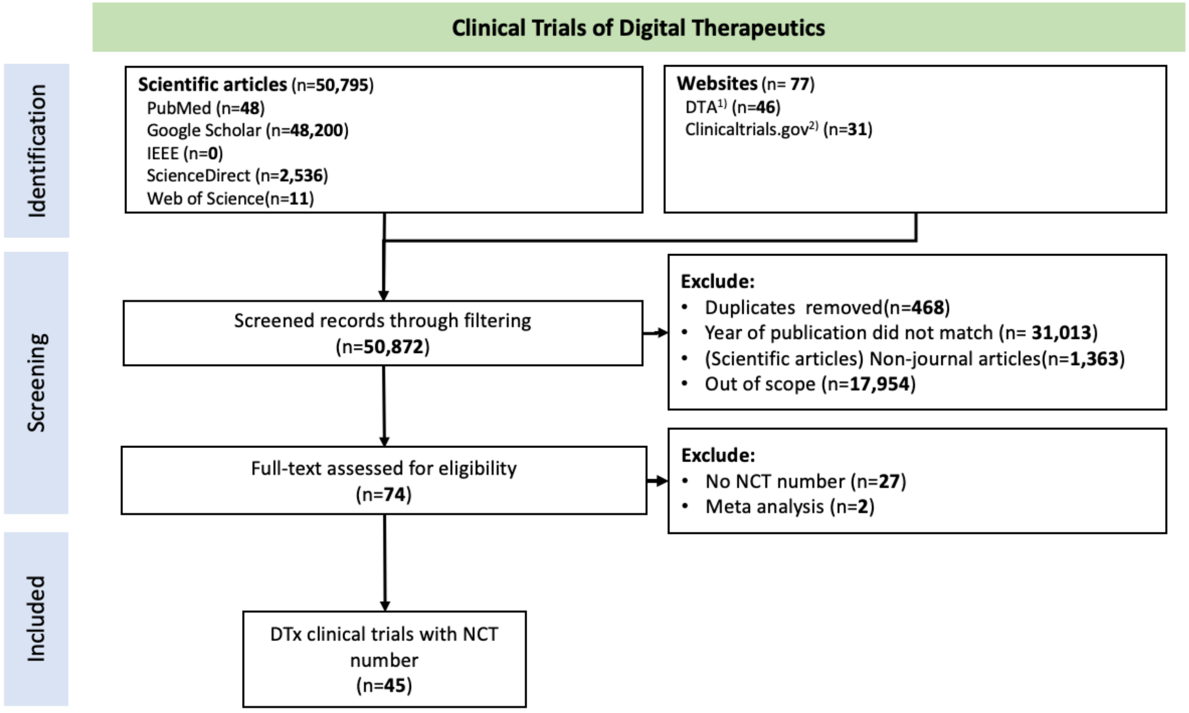

<Search string >
Scientific articles : (Digital therapeutics OR DTx) AND (FDA OR SaMD OR MDD OR MDR) AND (Digital health OR Healthcare) AND (Smartphone OR Application)
Clinicaltrials.gov website : "Digital therapeutics"

<Website list>
1) DTA https://dtxalliance.org/understanding-dtx/product-library/
2) Clinicaltrials.gov https://clinicaltrials.gov/ct2/results?cond=&term=%22digital+therapeutics%22&cntry=&state=&city=&dist=

<Abbreviation list>
DTA : Digital Therapeutics Alliance, **NCT** : The National Clinical Trial

Supplementary Figure 2. Flowchart for search process related to clinical trials of digital therapeutics

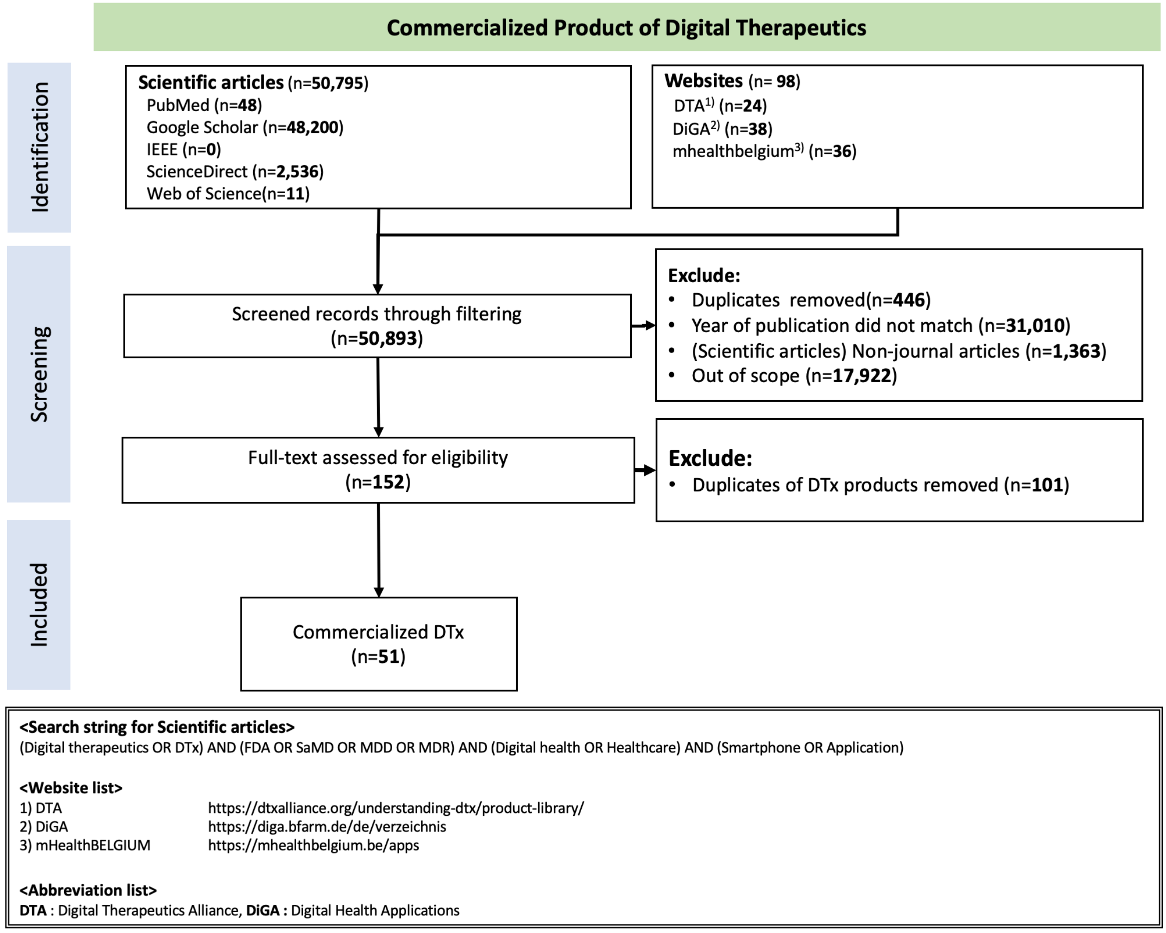

Supplementary Figure 3. Flowchart for search process related to commercialized DTx of digital therapeutics

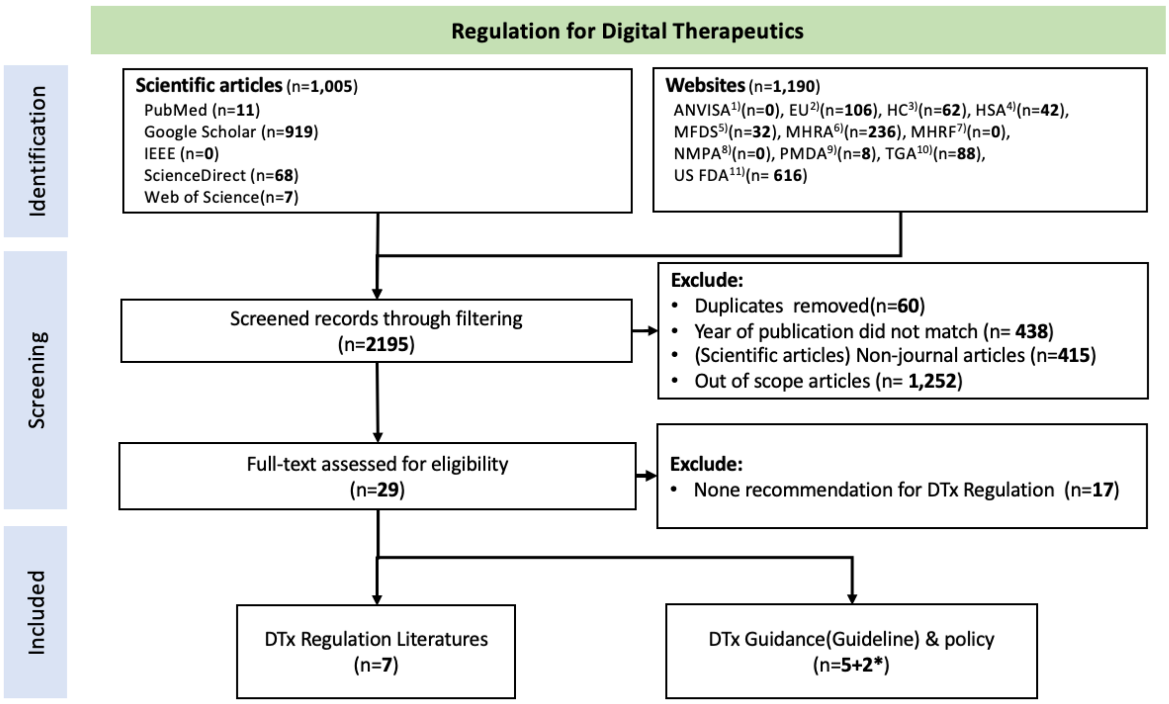

<Search string>

Scientific articles : ("Digital therapeutics" OR DTx) AND ("Regulation")

Websites : "Digital therapeutics" OR "DTx" OR "Guidance" OR "Guideline" OR "Policy."

<Website list>

1) ANVISA https://www.gov.br/anvisa/pt-br/english/regulation-of-products/medical-devices
2) EU https://health.ec.europa.eu/medical-devices-sector/new-regulations/guidance-mdcg-endorsed-documents-and-other-guidance_en
3) HC https://www.canada.ca/en/health-canada/services/drugs-health-products/medical-devices/application-information/guidance-documents.html
4) HSA https://www.hsa.gov.sg/medical-devices/guidance-documents
5) MFDS https://www.mfds.go.kr/eng/brd/m_40/list.do?page=3&srchFr=&srchTo=&srchWord=&srchTp=&itm_seq_1=0&itm_seq_2=0&multi_itm_seq=0&company_cd=&company_nm=
https://www.mfds.go.kr/brd/m_1060/list.do?multi_itm_seq=0&board_id=data0011&seq=&data_stts_gubun=C1004&srchTp=0&srchWord=
6) MHRA https://www.gov.uk/search/guidance-and-regulation?parent=medicines-and-healthcare-products-regulatory-agency&keywords=guidance&organisations%5B%5D=medicines-and-healthcare-products-regulatory-agency&order=relevance
7) MHRF https://minzdrav.gov.ru/en/poleznye-resursy
8) NMPA http://english.nmpa.gov.cn/medicaldevices.html
9) PMDA https://www.pmda.go.jp/english/review-services/regulatory-info/0002.html
10) TGA https://www.tga.gov.au/resources/resource?f%5B0%5D=topics%3A225&f%5B1%5D=type%3A201
11) US FDA https://www.fda.gov/medical-devices/device-advice-comprehensive-regulatory-assistance/guidance-documents-medical-devices-and-radiation-emitting-products

*During manuscript revision, two new DTx guidelines were published from the MFDS.

<Abbreviation list>

ANVISA : Brazilian Health Surveillance Agency (Brazil), **EU** : European Commission Directorate (EU), **HC** : Health Canada (Canada), **HSA** : Health sciences Authority (Singapore), **MFDS** : Ministry of Food and Drug Safety (Republic of Korea), **MHRA** : Medicines and Healthcare products Regulatory Agency (United Kingdom), **MHRF** : Ministry of Health of Russian Federation (Russia), **NMPA** : National Medical Products Administration (China), **PMDA** : Pharmaceutical and Medical Devices Agency (Japan), **TGA** : Therapeutic Goods Administration (Australia), **US FDA** : U.S. Food and Drug Administration (USA)

Supplementary Figure 4. Flowchart for search process related to regulation of digital therapeutics

Supplementary Table 1. Status of clinical trials with NCT number related on digital therapeutics registered on ClinicalTrials.gov (https://clinicaltrials.gov) or on DTA website (https://dtxalliance.org)

Conditions	Status	Interventions	Sponsor	Locations	Allocation	Enrollment (Age)	Intervention model	Primary purpose/outcomes	NCT number
Schizophrenia	Active, not recruiting	Device: CT-155	Click Therapeutics	US	N/A	48 (18–64)	Single Group Assignment	Primary purpose: Treatment<br>Primary outcome measures:<br>To explore feasibility and acceptability of the treatment monitoring and educational components of CT-155 app	NCT05438160
COPD	Unknown	Behavioral: Digital therapeutics	Senscio Systems		RCT	240 (All)	Parallel Assignment	Primary purpose: Supportive care<br>Primary outcome measures: Reduction in acute care utilization	NCT03131622
Drug use disorders, illicit drug use, alcohol-related disorders	Recruiting	Behavioral: Health coaching<br>Practice facilitation standard implementation	Kaiser Permanente	US	Non- RCT	700 (18, and older)	Parallel Assignment	Primary purpose: Health services research<br>Primary outcome measures:<br>Reach of the digital therapeutic to patients in the primary care clinic, Fidelity of patients use of the digital therapeutic to clinical recommendations	NCT04907045
Major depressive disorder	Not yet recruiting	Device: MEL-T01, MEL-S01	Aalto University	FI	RCT	800 (18–65)	Parallel Assignment	Primary purpose: Treatment<br>Primary outcome measures:<br>PHQ-9 total change from baseline to posttreatment: experimental group vs. treatment as usual group active comparator group vs. treatment as usual group<br>experimental group vs. active comparator group	NCT05426265
Autism spectrum disorder	Completed	Diagnostic Test: Cognoa ASD diagnostic device	Cognoa, Inc.	US	N/A	711 (18–72 months)	Single Group Assignment	Primary purpose: Diagnostic<br>Primary outcome measures: Positive and negative predictive value of ASD Dx in relation to clinician diagnostic evaluation, No result percentage	NCT04151290
Chronic low back pain, mastectomy, lumpectomy, migraine	Recruiting	Device: JOGO Digital Therapeutics EMG Biofeedback for Lower back pain, persistent post-mastectomy pain, migraine	Brigham and Women's Hospital	US	RCT	330 (18–65)	Parallel Assignment	Primary purpose: Treatment<br>Primary outcome measures:<br>Change in pain intensity after the treatment period	NCT04607460
System; lupus erythematosus	Complete	Behavioral: Mymee Program	Mymee, Inc.	US	RCT	50 (18, and older)	Parallel Assignment	Primary purpose: Treatment<br>Primary outcome measures:<br>Brief pain inventory-short form (BPI-SF) , Functional assessment of chronic illness therapy-Fatigue (FACIT-Fatigue), LupusQOL	NCT03426384
Heart failure	Recruiting	Other: Continuum HFC-IN<br>Other: Standard of care for the follow-up<br>Other: Continuum HFC-OUT	CRCHUM	CA	RCT	400 (18, and older)	Parallel Assignment	Primary purpose: health services research<br>Primary outcome measures:<br>CLIC IN ONLY: care consumption, CLIC OUT ONLY: change in guideline-directed medical therapy by classes, CLIC OUT ONLY: change in guideline-directed medical therapy by dose	NCT05377190

Postoperative pain	Recruiting	Device: RelieVRx headset<br>Drug: multi-modality pain management	AppliedVR, Inc.	US	RCT	113 (18, and older)	Parallel Assignment	Primary purpose: Supportive care<br>Primary outcome measures: Acute postoperative pain, Opioid consumption	NCT04010266
Chronic low back pain	Active, not recruiting	Other: DTx for pain, control software, standard care	Orion Corporation, Orion Pharma	FI	RCT	100 (18, and older)	Parallel Assignment	Primary purpose: Other<br>Primary outcome measures:<br>1) Self-report of disability, Oswestry, pain interface, 2) Objective report of disability, steps, 3) Fear of movement and re-injury, 4) Pain Behaviour, pain medications, 5) Quality of life, own experience, adverse events, change	NCT04225884
Multiple sclerosis,<br>mild cognitive impairment,<br>cancer	Recruiting	Other: CT-100 DiNaMo, Care-as-Usual	CCT Research	US	RCT	114 (22–75)	Parallel Assignment	Primary purpose: Basic Science<br>Primary outcome measures: Change in cognitive functioning<br>intervention group compared with a care-as-usual control group	NCT05438147
Cancer	Recruiting	Device: Attune, Cerena	www.restoreclinicaltrial.com	US	RCT	352 (18, and older)	Parallel Assignment	Primary purpose: Treatment<br>Primary outcome measures: Symptoms of anxiety	NCT05227898
Cognitive dysfunction, Covid-19	Recruiting	Device: AKL-T01	Weill Medical College of Cornell University	US	RCT	125 (18–89)	Crossover Assignment	Primary purpose: Treatment<br>Primary outcome measures: Change in cognitive function, as measured by the digit symbol matching task	NCT04843930
Diabetes 1, 2<br>Prediabetes	Unknown	Behavioral: One Drop | Experts on track, Experts on call, experts revive	Chandra Osborn	US	RCT	500 (18–75)	Parallel Assignment	Primary purpose: Treatment<br>Primary outcome measures: Within- and between-group changes in central lab-assessed hemoglobin A1c measured at baseline and 3 months.	NCT03459573
Fatigue, dyspnea, cognitive impairment, muscle pain	Enrolling by invitation	Device: the multi-domain Tai Chi Digital Therapy Software Application	Tim Shi	US	RCT	200 (18–75)	Parallel Assignment	Primary purpose: Supportive care<br>Primary outcome measures: Change in physical activity ability measured by 6-minute walking distance measurement (6MWT), Overall respiratory relief measured by the Post-COVID-19 Functional Status(PCFS) scale, Cognitive impairment improvement measured by the well-validated neuropsychological measurement tests	NCT05419219
Opioid, drug use disorders	Recruiting	Behavioral: Standard implementation<br>Behavioral: Health Coaching<br>Behavioral: Practice Facilitation	Kaiser Permanente	US	RCT	13,000 (18, and older)	Factorial Assignment	Primary purpose: Health services research<br>Primary outcome measures: Reach of the digital therapeutic to patients in the primary care clinic, Fidelity of patients' use of the digital therapeutic to clinical recommendations	NCT05160233
Substance use disorder	Complete	Behavioral: TES-NAV<br>Behavioral: TAU	New York State Psychiatric Institute	US	RCT	53 (18–99)	Single Group Assignment	Primary purpose: Treatment<br>Primary outcome measures: Consecutive weeks of drug/alcohol abstinence (12 weeks)	NCT03363256

Covid-19, dyspnea	Complete	Device: COVVR_Synch Device: COVVR_Asynch	Ecole Polytechnique Fédérale de Lausanne	SZ	RCT	26 (18, and older)	Crossover Assignment	Primary purpose: Supportive care Primary outcome measures: Efficacy (breathing comfort & discomfort), VR intervention feasibility	NCT04844567
Virtual reality	Recruiting	Device: VR training Device: VR activity	Medical University of Lodz	PL	RCT	100 (65–85)	Parallel Assignment	Primary purpose: Prevention Primary outcome measures: Working memory and Attention	NCT05369897
Insomnia, sleep disorder, sleep hygiene	Enrolling by invitation	Behavioral: Online Cognitive-Behavioral Therapy for Insomnia and complementary therapies	SleepUp Tecnologia em Saúde Ltda	BR	RCT	120 (20–60)	Parallel	Primary purpose: Treatment Primary outcome measures: Adherence to the treatment, Response to treatment, Remission of symptoms, and Maintenance of results	NCT04949360
Chronic pain, low back pain, shoulder pain	Complete	Device: telemedicine digital therapeutic	Karuna Labs Inc.	US	RCT	10 (18, and older)	Single Group Assignment	Primary purpose: Treatment Primary outcome measure: Disabilities of the arm, shoulder, and hand and Upper extremity functional index	NCT05375500
Pre-diabetes, Overweight and obesity	Unknown	Behavioral: MyDiPP (Malaysia Diabetes Prevention Program) Other: usual care	University Sultan Zainal Abidin	MY	RCT	100 (18–65)	Parallel Assignment	Primary purpose: Prevention Primary outcome measures: body weight	NCT03997656
Obesity, health behavior lifestyle	Not yet recruiting	Device: DTXO APP Device: PLACEBO APP - Control group	Advice Pharma Group Srl		RCT	246 (18–65)	Parallel Assignment	Primary purpose: Supportive care Primary outcome measures: Evaluation of change in body weight (kg).	NCT05394779
Post-partum depression	Recruiting	Device: Stella (TM) Mobile Application	Curio Digital Therapeutics, Inc.	US	N/A	65 (18–50)	Single Group Assignment	Primary purpose: Treatment Primary outcome measures: Change in level of depression as measured using Edinburgh postpartum depression (EPDS)	NCT05077644
Respiratory system disease, chronic obstructive pulmonary disease, asthma, COPD	Recruiting	Behavioral: Conventional rehabilitation treatment (e.g., leaflet) educated by hospital Device: Digital treatment based on Respiratory Rehabilitation Software	LifeSemantics Corp.	KR	RCT	100 (19–80)	Parallel Assignment	Primary purpose: Treatment Primary outcome measures: The 6-min walking distance (6-min walking distance, 6MWD) change amount (in meters) at 12 weeks after respiratory rehabilitation compared to the baseline	NCT05299385
Pulmonary disease, chronic obstructive pulmonary disease	Complete	Device: Kaia COPD Application Other: Usual care	Kaia Health Software	SZ	RCT	104 (40, and older)	Parallel Assignment	Primary purpose: Treatment Primary outcome measures: Steps per day as mean over one week from baseline until the treatment end	NCT04299165

Parkinson’s disease	Not yet recruiting	Device: Digital music therapeutic Behavioral: Active-Control	Boston University Charles River Campus	US	RCT	44 (40–80)	Parallel Assignment	Primary purpose: Treatment Primary outcome measures: Physical activity based on the amount of moderate intensity walking, Gait quality based on variability of stride length, Gait quality based on variability of swing time, Self-Report Behavioral Automaticity Index (SRBAI)	NCT05421624
Chronic kidney diseases, diabetes arterial hypertension (10 more)	Recruiting	Device: KidneYou App	Advice Pharma Group Srl	IT	RCT	210 (18, and older)	Parallel Assignment	Primary purpose: Supportive care Primary outcome measures: Evaluation of changes in azoturia, distance covered, and perceived Stress	NCT05286632
Multiple myeloma, solitary plasmacytoma, amyloidosis	Recruiting	Device: RITA (App)	Advice Pharma Group Srl	IT	Non-RCT	122 (18, and older)	Parallel Assignment	Primary purpose: Supportive care Primary outcome measures: Evaluating changes in actual dose intensity	NCT05260203
Cognitive decline	Recruiting	Device: CURATE.AI	National University Hospital	SG	N/A	15 (21–99)	Single Group Assignment	Primary purpose: Treatment Primary outcome measures: Patient acceptability of the digital cognitive test battery DI/DD obtained during a semi-structured interview, Patient adherence to the DI/DD, Patient attrition rate to the DI/DD, Percentage of CURATE.AI profiles successfully created and applied, Timely delivery of DI/DD at indicated time points, Digital intervention limited efficacy, and Digital diagnostic limited efficacy	NCT04848935
Dysarthria as late effect of stroke	Enrolling by invitation	Device: Mobile applçication Other: Treatment as usual	Ewha Womans University Seoul Hospital	KR	RCT	60 (All)	Parallel Assignment	Primary purpose: Treatment Primary outcome measures: Speech intelligibility	NCT05146765
ADHD	Completed	Device: AKL-T01	Akili Interactive Labs, Inc.	US	N/A	203 (8-14)	Single Group Assignment	Primary purpose: Treatment Primary outcome measure: Change in Impairment Rating Scale (Clinician Report) Overall Impairment; Cohort 1: Stimulant,	NCT03649074
Chronic Migraine	Completed	Device: Nerivio	Theranica	US	N/A	126 (18-75)	Single Group Assignment	Primary purpose: Treatment Primary outcome measure: Pain Relief at 2 Hours Post-treatment	NCT04194008
Depression	Completed	Device: Device: Limbix Spark Other: Psychoeducation Propeller Health System (formerly Asthmapolis System)	Limbix Health, Inc.	US	RCT	227 (13-21)	Parallel Assignment	Primary purpose: Treatment Primary outcome measure: Phase I - Feasibility (Eligibility), Number of Participants With Willingness to Participate and Program Adherence, Phase I - Feasibility (Satisfaction), Phase I - Feasibility (Safety), Phase II - Change in Depressive Symptoms	NCT04524598

Stress Urinary Incontinence	Completed	Device: Leva Pelvic Digital Health System<br>Other: Kegel exercises	Renovia, Inc.	US	RCT	369 (18-100)	Parallel Assignment	Primary purpose: Treatment<br>Primary outcome measure: Efficacy - Urogenital Distress Inventory (UDI-6), Efficacy - Bladder diaries	NCT04508153
Asthma	Completed	Device: Propeller Health System (formerly Asthmapolis System)	Reciprocal Labs	US	RCT	495 (5-90)	Parallel Assignment	Primary purpose: Treatment<br>Primary outcome measure: Change in Mean SABA Use	NCT01509183
Insomnia<br>Sleep Initiation and Maintenance Disorders	Completed	Behavioral: Internet Intervention<br>Behavioral: Patient Education Website	University of Virginia	US	RCT	303 (21-65)	Parallel Assignment	Primary purpose: Treatment<br>Primary outcome measure: Primary sleep symptoms: sleep onset latency (SOL), wake after sleep onset (WASO), insomnia severity (ISI)	NCT01438697
Diabetes Mellitus, Type 2<br>Hypoglycemia	Completed	Device: d-Nav<br>Device: Blood Glucose Monitoring System	Hygieia Research LLC	US	RCT	181 (21-70)	Parallel Assignment	Primary purpose: Treatment<br>Primary outcome measure: Reduction in HbA1C	NCT02424500
Panic Disorder	Completed	Device: Canary Breathing System	Palo Alto Health Sciences, Inc.	US	N/A	69 (18-60)	Single Group Assignment	Primary purpose: Treatment<br>Primary outcome measure: Percent of Subjects Achieving a 40% Decrease in Overall PDSS Score (Clinically Significant Response)	NCT01955954
Stress Disorders, Post-Traumatic	Completed	Device: Freespira Breathing System	Palo Alto Health Sciences, Inc.		N/A	55 (18, and older)	Single Group Assignment	Primary purpose: Treatment<br>Primary outcome measure: Primary outcome of this study will be quantitative improvements using the Clinician Administered PTSD Scale (CAPS - 5)	NCT03039231
Acute Migraine	Terminated	Device: Nerivio	Theranica	US	N/A	60 (12-17)	Single Group Assignment	Primary purpose: Treatment<br>Primary outcome measure: Safety of Nerivio Device	NCT04089761
Migraine Without Aura<br>Migraine With Aura	Completed	Device: Nerivio Migra-1 active device<br>Device: Nerivio Migra-1 Sham device	Theranica	US	RCT	296 (18-75)	Parallel Assignment	Primary purpose: Treatment<br>Primary outcome measure: Percentage of Participants With Reduction of Migraine Headache at 2 Hours Post Treatment	NCT03361423
Migraine Headache	Completed	Device: Randomized treatment/placebo delivery of transcutaneous electro stimulation via a dedicated device Avital.	Theranica	IL	N/A	87 (18-75)	Single Group Assignment	Primary purpose: Treatment<br>Primary outcome measure: Test of Variables of Attention-Attention Performance Index (Change From Baseline to Posttreatment)	NCT02453399
Attention Deficit Disorder With Hyperactivity	Completed	Device: AKL-T01<br>Device: AKL-T09	Akili Interactive Labs, Inc.	US	RCT	348 (8-12)	Parallel Assignment	Primary purpose: Treatment<br>Primary outcome measure: Test of Variables of Attention-Attention Performance Index (Change From Baseline to Posttreatment)	NCT02674633

Cancer	Recruiting	Device: Electronic patient reported outcomes	Oulu University Hospital	FI	N/A	40 (18, and older)	Single Group Assignment	Primary purpose: Supportive care<br>Primary outcome measure: Change in the spectrum of patient reported symptoms, Change in Patient reported symptom severity, Change in the number of triggered alerts by the tool, Changes in Quality of Life according to QLQ-C30 Summary scores, Correlation between changes in different symptoms and their severity to treatment side-effects, cancer progression, other medical events, or survival, Changes in Patient compliance Questionnaire, Changes in patient compliance according to answering rate to symptom questionnaires, Change in patient compliance according to answering rates to QLQ-C30 questionnaire, Correlation of change in baseline Hb values compared to control Hb values to treatment side-effects, cancer progression, other medical events or survival, Correlation of change in baseline leucocyte values compared to control leucocyte values to treatment side-effects, cancer progression, other medical events or survival, Correlation of change in baseline lymphocyte values compared to control lymphocyte values to treatment side-effects, cancer progression, other medical events or survival, Correlation of change in baseline neutrophil values compared to control neutrophil values to treatment side-effects, cancer progression, other medical events or survival, Correlation of change in baseline CRP values compared to control CRP values to treatment side-effects, cancer progression, other medical events or survival	NCT03928938

Supplementary Table 2. Types of commercialized digital therapeutics with regulatory approval[1-4]

Product	Company	Disease	Characteristic	Approval status
WellDoc	BlueStar	Type 1, 2 diabetes	Diabetes self-management app	FDA-510(k)
Voluntis	Insulia	Type 1, 2 diabetes	Insulin dosage calculation app for medication management	FDA-510(k), EU-CE Mark
Diabeo	Voluntis	Type 1, 2 diabetes	Diabetes mobile app	EU-CE Mark
d-Nav	HYGIEIA	Type 1, 2 diabetes	Insulin Management Program	FDA-510(k)
mySugr	Roche Diagnostics Belgium NV	Type 1, 2 diabetes	Diabetes management app	EU-CE Mark
Dario	LabStyle Innovations	Type 1, 2 diabetes	Cloud-based diabetes management system	FDA-510(k)
ESYSTA App & Portal	Emperra GmbH E-Health Technologies	Type 1, 2 diabetes	Diabetes management app	EU-CE Mark
CardiacSense	Arseus Hospital NV	Heart disease	Heart disease monitoring app	EU-CE Mark
CureApp-SC	CureAPP, Inc.	Smoking cessation	Smoking cessation monitoring mobile app	MHLW (JP)
reset	Pear Therapeutics	Substance use disorder (SUD)	Computerized behavioral therapy mobile app	FDA-510(k)
reSET-O	Pear Therapeutics	Opioid use disorder (OUD)	Computerized behavioral therapy mobile app	FDA-510(k)
vorvida	Orexo	Alcohol use	Alcohol use management app	FDA COVID-19 Public Health Emergency Guidance
Freespira	Palo Alto Health Sciences	Panic disorder and post-traumatic stress disorder (PTSD)	Stress relaxation biofeedback device for PTSD/panic disorder patients	FDA-510(k)
Natural Cycles	Natural Cycles	Birth control	Mobile app	FDA-De Novo, EU-CE Mark
Propeller Health	ResMed (Propeller Health)	Asthma, COPD	Chronic obstructive pulmonary disease management app	FDA-510(k)
Nucleus Smart App	Cochlear	Ear disorder	Cochlear implant management app	EU-CE Mark
ProAir Digihaler	Teva	Asthma, COPD	COPD management app	FDA-510(k)
EndeavorRx	Akili Interactive Labs	Pediatric ADHD	Video game	FDA-De Novo, EU-CE Mark
TALi	TALi Digital	Attention impairment	Mobile-based game	PTY 510(k) exempt, EU-CE Mark
Somryst	Pear Therapeutics	Chronic insomnia	Insomnia and sleep restriction management mobile app	FDA-510(k)

Oleena	Voluntis	Cancer	Oncology-related symptoms management mobile app	FDA-510(k)
PRO-React Onco	CANKADO	Cancer	Breast cancer management app	EU-CE Mark
Kaiku Health	Kaiku Health	Cancer	Cancer care app	EU-CE Mark
Noona	Varian Medical Systems Belgium	Cancer	Cancer care app	EU-CE Mark
RemeCare	Remecare	Cancer	Cancer care app	EU-CE Mark
Sleepio	Big Health	Sleep disorder	Psychiatric disorder mobile app	EU-CE Mark
Airview	ResMed	Sleep disorder	Sleep disorder breathing management app	EU-CE Mark
Care Orchestrator	Philips Belgium Commercial	Sleep disorder	sleep apnea monitoring app	EU-CE Mark
HelloBetter Diabetes and Depression	GET.ON Institut für Online Gesundheitstrainings GmbH	Depression	Depression management app in diabetes patients	EU-CE Mark
HelloBetter Chronic Pain	GET.ON Institut für Online Gesundheitstrainings GmbH	Chronic pain	Chronic pain management app	EU-CE Mark
HelloBetter Stress and Burnout®	GET.ON Institut für Online, Gesundheitstrainings GmbH	Stress and burnout	Stress management app	EU-CE Mark
HelloBetter Vaginismus Plus	GET.ON Institut für Online, Gesundheitstrainings GmbH	Vaginismus, dyspareunia and genitopelvic pain/penetration disorder (GPPPD)	Genito-pelvic pain/penetration disorder (GPPPD) management app	EU-CE Mark
leva	Renovia, Inc.	Urinary incontinence	urinary incontinence management app	FDA-510(k)
Cara Care	HiDoc Technologies	irritable bowel syndrome	irritable bowel syndrome care app	EU-CE Mark
Nerivio	Theranica	Migraine	Migraine management app	FDA-De Novo, EU-CE Mark
moveUP Coach	moveUP	hip and knee arthroplasty	hip and knee arthroplasty treatment, rehabilitation app	EU-CE Mark

FDA-510(k): Applies to premarket notification and Class 1 and Class 2 medical devices. FDA-de novo: Abbreviation for Evaluation of Automatic Class III Designation. It is a new concept medical device without product classification and is a special expedited approval system granted when the safety of the technology is found to be above a certain level. EU-CE Mark: Products that meet all requirements of European standards relating to safety, health, environment, and consumer protection. Medical devices in Europe must be CE certified. MHLW: Medical device products approved by the Ministry of Health, Labor and Welfare of Japan

Supplementary Table 3. Major indications of digital therapeutics published in peer-reviewed journals and posted on the DTA product library website[1,3,5,6]

Category	Chronic disease	Drug abuse	Psychiatry	Sleep	Cancer	Cognitive	Obstetrics and gynecology	Immune disease	Cranial nerve	Etc.
Indications	Type 1 and 2 diabetes, asthma, COPD<br><br>Chronic pain<br><br>Respiratory system disease (Chronic obstructive pulmonary disease, asthma COPD, pulmonary disease, chronic obstructive)<br><br>Chronic low back pain<br><br>Chronic Kidney Diseases<br><br>Arterial Hypertension<br><br>diabetes (type l and ll) Fatigue<br><br>(chronic) heart failure	Smoking cessation<br><br>Substance use disorder (SUD)<br><br>Opioid use disorder (OUD)<br><br>Alcohol use Drug use disorder with Alcohol and Illicit substance use disorder (SUD)	Panic disorder and post-traumatic stress disorder (PTSD)<br><br>Pediatric ADHD<br><br>Attention impairment<br><br>Generalized anxiety disorder<br><br>Depression<br><br>Stress and burnout<br><br>Schizophrenia<br><br>Major depressive disorder<br><br>Post-partum Depression	Chronic insomnia<br><br>Sleep disorder<br><br>Sleep disorder (Insomnia, sleep Hygiene)	All cancer Cancer<br><br>Mastectomy<br><br>Lumpectomy<br><br>Amyloidosis<br><br>Multiple Myeloma,<br><br>Solitary Plasmacytoma	Cognitive decline, cognitive dysfunction<br><br>Mild cognitive impairment	Vaginismus, dyspareunia, and genitopelvic pain/penetration disorder (GPPPD)<br><br>Birth control	Lupus Erythematosus<br><br>Multiple Sclerosis	Migraine<br><br>Parkinson's Disease<br><br>Dysarthria as late effect of stroke	(Gastroenterology) irritable bowel syndrome<br><br>(Musculoskeletal disease) hip and knee arthroplasty<br><br>(Otorhinolaryngology) Ear disorder<br><br>(Epidemic) Covid-19<br><br>(Respiratory system disease) Dyspnea<br><br>(Urination disease) Urinary incontinence

Supplementary Table 4. Core technology of digital therapeutics

Company	Product	Key technical elements	Interface
Pear Therapeutics	reSET	· Mobile app<br>· Content	· Smartphone application
	reSET-O		
	Somryst		
	PEAR-004		
Voluntis	Insulia	· Mobile app<br>· Web-app<br>· Content	· Smartphone application<br>· Web application
	Oleena		
	eCO		
	Theraxium Oncology program		
WellDoc	BlueStar	· Mobile app<br>· Web app<br>· Content	· Smartphone application<br>· Web application
Propeller Health	Nebulizer	· Medical device<br>· Mobile app	· IoT device<br>· Smartphone application
MindMaze	Mind Motion PRO	· VR<br>· Game content	· Wearable Device<br>· IoT-based game application
	Mind Motion GO		
Palo Alto Health Sciences	Freespira	· Mobile app<br>· Content	· Smartphone application
Akili Interactive	EndeavorRx	· Game<br>· Content	· IoT Device<br>· Smartphone application
	AKL-T02, AKL-T03		
Big Health	Sleepio	· Mobile app<br>· Content	· Smartphone application
Omada Health	Omada	· Mobile app<br>· Content	· Smartphone application
Kaia Health	COPD Exercise App	· Mobile app<br>· AI<br>· Content	· Smartphone application
	Musculoskeletal Pain Management App		
Cognoa	Autism, ADHD, Pediatric insomnia, Anxiety Therapeutic	· Mobile app<br>· Content	· Smartphone application
Click Therapeutics	Clikotine	· Mobile app<br>· Web app<br>· Content	· Smartphone application<br>· Web application
	CT-141(Clickadian), CT-152, CT-111	· Mobile app<br>· Content	· Smartphone application
ATENTIV	ATENTIVmynd	· Mobile app<br>· Game, BCI<br>· Content	· Smartphone/tablet application
Teva	ProAir Digihaler	· Sensor<br>· Mobile app<br>· Content	· IoT device<br>· Smartphone application
Dthera Sciences	DTHR-ALZ	· Mobile app<br>· AI<br>· Content	· Smartphone application
Susmed	Yawn	· Mobile app<br>· Content	· Smartphone application
Vivid Vision	Vivid Vision	· VR<br>· Game<br>· Content	· IoT device<br>· IoT-based game application
Limbix	Limbix Spark	· Mobile app<br>· Content	· Smartphone application
	Limbix VR	· VR<br>· Content	· IoT Device<br>· Smartphone/PC application
Proteus Digital Health	Ingestible Event Marker (IEM)	· Mobile app<br>· Web app<br>· Sensor<br>· Patch	· Wearable device<br>· Smartphone application<br>· Web application
Otsuka	Abilify Mycite	· Mobile app<br>· Web app<br>· Sensor<br>· Patch	· Smartphone application<br>· Web application<br>· Wearable device

BCI: Brain Computer Interface; IoT: Internet-of-Things; VR: Virtual Reality

Supplementary References

1 Hong, J. S., Wasden, C. & Han, D. H. Introduction of digital therapeutics. *Comput Methods Programs Biomed* **209**, 106319, doi:10.1016/j.cmpb.2021.106319 (2021).

2 Patel, N. A. & Butte, A. J. Characteristics and challenges of the clinical pipeline of digital therapeutics. *NPJ Digit Med* **3**, 159, doi:10.1038/s41746-020-00370-8 (2020).

3 Digital Therapeutics Alliance. *Product Library*, <https://dtxalliance.org/understanding-dtx/product-library/> (2022).

4 Chung, J. Y. Digital therapeutics and clinical pharmacology. *Transl Clin Pharmacol* **27**, 6-11, doi:10.12793/tcp.2019.27.1.6 (2019).

5 National Library of Medicine, U. S. *Indications type of digital therapeutics in clinicaltrials.gov*, <https://clinicaltrials.gov/ct2/results?cond=&term=%22digital+therapeutics%22&cntry=&state=&city=&dist=> (2022).

6 Tye-Murray, N., Spehar, B., Mauze, E. & Cardinal, C. Hearing Health Care Digital Therapeutics: Patient Satisfaction Evidence. *Am J Audiol* **31**, 905-913, doi:10.1044/2022_aja-21-00236 (2022).
